# Illuminating Progress: A Comprehensive Review of the Evolution of Phototherapy for Neonatal Hyperbilirubinemia

**DOI:** 10.7759/cureus.55608

**Published:** 2024-03-05

**Authors:** Ankita Patel, Jayant D Vagha, Revat J Meshram, Amar Taksande, Rahul Khandelwal, Aditya Jain, Astha Khurana

**Affiliations:** 1 Pediatrics, Jawaharlal Nehru Medical College, Datta Meghe Institute of Higher Education and Research, Wardha, IND; 2 Pediatrics, awaharlal Nehru Medical College, Datta Meghe Institute of Higher Education and Research, Wardha, IND

**Keywords:** pediatric care, phototherapy modalities, newborn jaundice, bilirubin metabolism, phototherapy, neonatal hyperbilirubinemia

## Abstract

This comprehensive review thoroughly examines the historical evolution, physiological foundations, and contemporary advancements in the application of phototherapy for neonatal hyperbilirubinemia. Neonatal hyperbilirubinemia, a common condition resulting from the immature hepatic processes in newborns, poses potential risks, including neurotoxicity, if left untreated. The review traces the historical progression from early recognition of neonatal jaundice to the development of various phototherapy modalities, showcasing the dynamic landscape of neonatal care. Emphasizing the physiological intricacies of bilirubin metabolism in neonates, the study underscores the vulnerability of newborns to hyperbilirubinemia due to delayed hepatic maturation. Phototherapy is a cornerstone in managing hyperbilirubinemia, demonstrating consistent efficacy in reducing unconjugated bilirubin levels. The implications for clinical practice are significant, offering healthcare professionals insights into tailoring treatment strategies based on individual neonatal characteristics and the severity of jaundice. Integrating advanced monitoring and control systems enhances the precision and safety of phototherapy. Recommendations for future research emphasize the need to investigate long-term outcomes, explore adjunctive therapies, and address resource limitations to ensure global access to effective neonatal care. Overall, this review contributes to the ongoing refinement of neonatal care practices, offering a comprehensive understanding of neonatal hyperbilirubinemia and its evolving treatment landscape.

## Introduction and background

Neonatal hyperbilirubinemia, characterized by elevated levels of unconjugated bilirubin in the blood of newborns, remains a common and concerning health issue affecting infants worldwide. Jaundice, the visible manifestation of hyperbilirubinemia, is particularly prevalent among neonates and is a result of the immature hepatic processes responsible for bilirubin metabolism. Despite being a physiological phenomenon in many cases, excessive bilirubin levels pose potential risks, including neurotoxicity, which can lead to long-term developmental complications if left untreated [1]. Understanding the physiological basis of neonatal hyperbilirubinemia is crucial for effective management. Bilirubin, a yellow pigment derived from the breakdown of hemoglobin, is typically conjugated in the liver and excreted in bile. However, neonates often exhibit a delayed maturation of hepatic function, leading to reduced bilirubin conjugation and excretion. This physiological vulnerability contributes to the increased susceptibility of newborns to hyperbilirubinemia [2].

Phototherapy has emerged as a cornerstone in the management of neonatal hyperbilirubinemia. The application of specific wavelengths of light facilitates the photoisomerization of unconjugated bilirubin in the skin, rendering it more water-soluble and amenable to excretion. This non-invasive and relatively simple therapeutic approach has proven effective in reducing serum bilirubin levels, thereby mitigating the risk of bilirubin-induced neurotoxicity [3]. This comprehensive review aims to delve into the historical evolution, physiological underpinnings, and contemporary advancements in phototherapy for neonatal hyperbilirubinemia. By synthesizing existing knowledge and exploring recent developments, the review seeks to provide a thorough understanding of the mechanisms, types, and efficacy of phototherapy. Additionally, it aims to address challenges, highlight innovations, and outline potential future directions in the field. Through this exploration, the review endeavors to contribute to the broader understanding of neonatal care and inform healthcare professionals, researchers, and policymakers about the current state and potential advancements in the treatment of neonatal hyperbilirubinemia.

## Review

Historical perspective

Early Recognition of Neonatal Jaundice

The historical acknowledgment of neonatal jaundice traces its origins to ancient times, with the earliest documented reference believed to be from a Chinese textbook approximately 1000 years ago [4]. However, it wasn't until the 18th and 19th centuries that medical theses, essays, and textbooks began delving into discussions regarding the causes and treatment of neonatal jaundice [4]. Over the years, the understanding of this condition has evolved, encompassing both severe and milder forms [5]. In the late 1950s, the development of phototherapy marked a significant milestone and is now widely acknowledged as the primary approach for managing neonatal jaundice [6]. The early identification of neonatal jaundice is paramount for timely and appropriate intervention. A study involving expectant mothers has underscored the crucial role of enhancing knowledge to facilitate the early recognition of neonatal jaundice [7]. This emphasizes the importance of educating both caregivers and healthcare providers about the signs and symptoms of neonatal jaundice to ensure prompt and effective management.

Initial Treatment Approaches

The treatment landscape for neonatal jaundice has undergone significant evolution over time. Exchange transfusion (ET) initially emerged as the first successful treatment for jaundice, holding a pivotal role as the primary intervention for severe unconjugated hyperbilirubinemia. However, contemporary medical practices have repositioned ET as a second-line treatment, with phototherapy now established as the primary therapeutic approach for unconjugated hyperbilirubinemia [5]. Developed in the late 1950s, phototherapy has become the cornerstone in managing neonatal jaundice [6]. This modality takes precedence in treating unconjugated hyperbilirubinemia, impacting approximately 50% of term and 80% of preterm infants [8]. Phototherapy is commonly employed to lower levels of unconjugated bilirubin, thereby preventing the onset of acute or chronic bilirubin encephalopathy [8]. In the majority of cases, phototherapy stands as the predominant medical treatment for hyperbilirubinemia in neonates [9]. It is crucial to recognize that the effectiveness of phototherapy may be contingent on variables such as the intensity of the light source, the total light dose administered, and other influencing factors [8]. The shift from exchange transfusion to phototherapy as the primary treatment for unconjugated hyperbilirubinemia reflects advancements in medical understanding and technological innovations for effectively managing this prevalent neonatal condition [8].

Development of Phototherapy as a Treatment Modality

Phototherapy has evolved into a significant treatment modality, extending its impact beyond neonatal jaundice to encompass various dermatological conditions. This therapeutic approach involves the controlled administration of non-ionizing radiation to the skin. It has effectively addressed several skin diseases, including parapsoriasis, psoriasis, eczema, and vitiligo [10]. Notably, the versatility of phototherapy extends beyond standalone treatment, as it is often employed with topical or systemic medications to enhance disease control [11]. The developmental trajectory of phototherapy has been shaped by its initial recognition of efficacy in treating infants with hyperbilirubinemia, resulting in a significant decrease in the reliance on exchange transfusion as a primary intervention [12]. Moreover, phototherapy has garnered recognition for being a safe, straightforward, and effective treatment modality, offering advantages such as lower costs and fewer side effects in managing various dermatological diseases [10,11]. This expansion of phototherapy's applications underscores its versatility and role as a valuable tool in the broader landscape of dermatological care.

Mechanism of phototherapy

Interaction Between Light and Bilirubin

The mechanism underlying phototherapy for neonatal hyperbilirubinemia is rooted in the interaction between light and bilirubin. When an infant's skin is exposed to light, the non-polar unconjugated bilirubin present in the skin undergoes a process known as photoisomerization. This transformation results in the conversion of bilirubin into water-soluble isomers that can be readily excreted from the body through bile or urine, consequently reducing serum bilirubin levels [13]. The efficacy and speed of this photoisomerization process are significantly influenced by the dose of phototherapy, a metric determined by factors such as the wavelength of the light, its intensity, and the distance between the light source and the infant [3]. Notably, light in the blue region of the spectrum, particularly around 460 nm, is highly absorbed by bilirubin, rendering it particularly effective for phototherapy [3]. Furthermore, under the influence of phototherapy, bilirubin undergoes photoisomerization at the meso double-bond, forming less lipophilic compounds that facilitate its efficient excretion from the body [14]. This detailed understanding of the phototherapy mechanism sheds light on the precise factors influencing its effectiveness in mitigating neonatal hyperbilirubinemia.

Photodegradation of Unconjugated Bilirubin

The photodegradation of unconjugated bilirubin entails the conversion of non-polar unconjugated bilirubin into water-soluble isomers through photoisomerization upon exposure to light. Extensive research has elucidated that the photodegradation rate of unconjugated bilirubin is intricately influenced by factors such as the light source, wavelength, and intensity. In vitro studies have indicated explicitly that the rate of photodegradation of unconjugated bilirubin surpasses that of other bilirubin subfractions, nearly doubling that of monoconjugated delta bilirubin, thus rendering it exceptionally susceptible to photoirradiation [15]. Furthermore, the photodegradation of bilirubin may result in the generation of photoproducts, including lumirubin, which have been scrutinized for their potential neuro-inflammatory effects [16]. Spectrophotometric characteristics of bilirubin undergo alterations induced by light irradiation, offering insights into the kinetics of bilirubin photodegradation [17]. Notably, the photodegradation of bilirubin has been associated with changes in the physical and chemical properties of DNA when exposed to light in its presence [18]. Additionally, under the influence of phototherapy, bilirubin undergoes photoisomerization at the meso double-bond, generating less lipophilic forms that facilitate efficient excretion from the body [14]. These multifaceted findings underscore the intricate nature of the photodegradation of unconjugated bilirubin, revealing potential implications that remain subjects of ongoing research.

Effects on Bilirubin Excretion

Phototherapy is a highly effective method for mitigating elevated serum bilirubin levels in neonatal hyperbilirubinemia. The phototherapy mechanism is centered on converting non-polar unconjugated bilirubin in the skin into water-soluble isomers, facilitating their excretion from the body through bile or urine and consequently reducing serum bilirubin levels [19,20]. Additionally, phototherapy contributes to an overall increase in bilirubin excretion by converting it to oxidation products [19]. Despite its efficacy, phototherapy is not without potential drawbacks. Excessive bilirubin decomposition induced by phototherapy may stimulate the intestinal wall, leading to unintended consequences [13]. Furthermore, the skin's protective barrier can be compromised during phototherapy, resulting in adverse effects such as burns, retinal damage, thermoregulatory instability, loose stools, dehydration, and skin irritation [20]. As such, while phototherapy remains a pivotal and commonly employed intervention, its application necessitates careful consideration of potential adverse effects and close monitoring to ensure the overall well-being of neonates undergoing treatment.

Types of phototherapies

Conventional Phototherapy

Conventional phototherapy, a modality employed in the treatment of neonatal hyperbilirubinemia, utilizes compact fluorescent lamps (CFLs) or halogen lamps [21]. While the irradiance of the light in conventional phototherapy is lower than that in intensive phototherapy, the actual values can vary significantly among different manufacturers [22]. In this approach, the infant is positioned under the light source, and the emitted light is absorbed by the skin, thereby converting bilirubin into a water-soluble form that can be excreted through urine and stool [11]. Often integrated with other treatments, such as topical or systemic medications, conventional phototherapy is utilized to enhance disease control [11]. However, akin to any therapeutic intervention, it is not without potential side effects, including erythema and burns. Vigilance is therefore crucial to detect and address any possible adverse events during treatment [11]. Despite reported satisfaction from users, the accessibility to phototherapy units emerges as a notable limiting factor for the widespread adoption of this therapy [11]. This recognition highlights the importance of considering logistical aspects in addition to therapeutic efficacy when evaluating the overall feasibility and impact of conventional phototherapy in neonatal care.

Intensive/High-Intensity Phototherapy

Intensive or high-intensity phototherapy is a treatment modality characterized by the application of elevated levels of spectral irradiance, typically defined as at least 30 µW/cm2 per nm, measured on the patient's skin. This approach finds utility in diverse medical applications, prominently in the treatment of neonatal jaundice and various skin conditions. The effectiveness of phototherapy hinges on several factors, including the wavelength of light, spectral irradiance, and the cumulative dose of light received. Intensive phototherapy has demonstrated superior efficacy in swiftly reducing bilirubin levels in newborns with jaundice compared to low-dose conventional phototherapy [22-24]. Ensuring the effectiveness and safety of intensive phototherapy requires meticulous measurement and monitoring of spectral irradiance [22]. This emphasis on precision and monitoring underscores the importance of balancing therapeutic effectiveness and safety considerations in applying intensive phototherapy for neonatal care and skin-related conditions.

Fiber-Optic Phototherapy

Fiber-optic phototherapy represents a specific type of phototherapy designed for treating infant jaundice, or hyperbilirubinemia, utilizing a portable device known as a fiber-optic biliblanket [25]. This innovative device comprises an illuminator, a fiber-optic pad, and a disposable pad cover [8,25]. The fiber-optic pad administers blue or white light directly onto the infant's skin from the illuminator, ensuring that the therapeutic light is precisely delivered to the skin without environmental contamination, enhancing patient and caregiver comfort [8,25]. Comparative studies have indicated that fiber-optic phototherapy yields results akin to blue light conventional phototherapy regarding bilirubin reduction rate and treatment duration. Notably, it surpasses the efficacy of conventional white light phototherapy [25,26]. One distinct advantage of fiber-optic phototherapy lies in its ability to minimize the risk of overheating in newborns, positioning it as the most advanced treatment option for newborn jaundice regarding safety and comfort [25]. This underscores the significance of technological advancements in improving the therapeutic landscape and ensuring optimal care for infants with hyperbilirubinemia.

Light-Emitting Diode (LED) Phototherapy

Phototherapy encompasses various modalities tailored to address diverse skin conditions and therapeutic objectives. One prominent approach is LED light therapy, widely employed for treating a spectrum of skin concerns, including acne, fine lines, and psoriasis [27]. LED light therapy manifests in different types, such as red-light and blue-light LED therapy, with some applications utilizing a combination of these wavelengths for enhanced efficacy [27]. Red light LED therapy is recognized for its potential to promote skin healing and reduce inflammation. In contrast, blue light LED therapy is often employed to target acne-causing bacteria and manage skin conditions with antimicrobial effects. In contrast, traditional phototherapy leverages ultraviolet (UV) light to address various skin conditions. This category includes diverse types, such as broad-band UVB, UVA, and PUVA therapy [28]. Broadband UVB therapy is commonly used to treat conditions like psoriasis, vitiligo, and eczema, while UVA therapy may be applied for certain skin disorders and in conjunction with photochemotherapy. PUVA therapy combines UVA exposure with the administration of a photosensitizing medication, serving as an effective intervention for certain skin disorders, such as psoriasis. Beyond skin-related applications, bright light therapy emerges as a distinct form of phototherapy for managing mood and sleep disorders [29]. This therapeutic approach involves exposure to intense light, often mimicking natural sunlight, to regulate circadian rhythms and alleviate symptoms associated with conditions like seasonal affective disorder and sleep disorders. The diverse range of phototherapies underscores the versatility of light-based interventions in medical and dermatological practices, showcasing the evolving landscape of treatments tailored to specific conditions and patient needs.

Home Phototherapy (HPT) Devices

HPT units have become increasingly available for the self-treatment of specific skin conditions, providing a range of options from hand-held and table-top units for localized treatment to cabinet or "walk-in" units designed for comprehensive full-body therapy [30]. Notable companies in this sector, such as Daavlin, UVABiotek, Solarc Systems Inc., and National Body Corp, offer diverse HPT solutions [30]. This form of treatment offers a non-drug alternative for individuals seeking the convenience and comfort of addressing their skin conditions within the familiar surroundings of their own homes [31]. The versatility of HPT units allows patients to choose devices that suit the size and scope of the affected areas, providing targeted treatment for specific skin concerns. From spot treatment for small areas to full-body therapy options, these devices empower individuals to tailor their treatment based on their unique needs. Despite the convenience and accessibility of HPT, it is imperative to use regulated devices and adhere to the guidance provided by healthcare providers to ensure the safe and practical application of these treatments [30]. This emphasizes the importance of responsible use, underlining the necessity of medical supervision to optimize treatment outcomes and mitigate potential risks associated with HPT. As technology continues to advance, HPT units represent an evolving facet of dermatological care, offering patients greater autonomy and flexibility in managing their skin conditions.

Efficacy and safety

Clinical Effectiveness of Phototherapy

Phototherapy emerges as a versatile and effective treatment modality across diverse age groups and skin conditions. Firstly, in the realm of atopic dermatitis, studies have demonstrated the efficacy of phototherapy for both acute cases (utilizing UVA1) and chronic cases (employing NB-UVB) in both adults and children [32]. Notably, the safety profile of phototherapy is generally favorable, characterized by a low incidence of short-term and long-term adverse effects. Neonatal jaundice represents another domain where phototherapy is commonly employed, proving effective in reducing the appearance of symptoms associated with psoriasis and eczema in newborns [21]. This underscores its significance as a therapeutic intervention in neonatal care. For the elderly population, phototherapy has exhibited effectiveness and reliability, with dedicated studies evaluating its impact on various skin disorders and quality of life within this age group [33]. The positive outcomes highlight the potential of phototherapy as a valuable component in the dermatological care of elderly individuals.

Furthermore, emerging research has explored the benefits of blue-green light therapy as a specific form of phototherapy, revealing its effectiveness in treating certain conditions [34]. This broad spectrum of applications underscores the versatility of phototherapy in addressing diverse skin-related concerns. Phototherapy stands as a proven and generally well-tolerated treatment modality for various skin conditions, including atopic dermatitis, neonatal jaundice, and skin disorders in the elderly. Despite its recognized safety, potential limitations such as costs, availability, and the need for specialized staff should be considered when contemplating phototherapy as a treatment option. This holistic understanding encourages healthcare providers and patients to weigh the benefits and considerations for optimal decision-making in adopting phototherapy into their treatment plans.

Adverse Effects and Concerns

The effectiveness and safety of phototherapy in managing neonatal jaundice have been firmly established. Phototherapy stands as the primary approach for addressing neonatal jaundice, contributing significantly to a reduction in the necessity for exchange transfusions - previously deemed challenging and fraught with risks [6]. Numerous studies have substantiated the efficacy and safety of phototherapy across physiological and pathological jaundice, encompassing HPT, integral phototherapy, and even phototherapy utilizing filtered sunlight [35-37].

These investigations underscore phototherapy's role as a reliable and secure treatment for infants experiencing jaundice, with notable implications such as diminished parental anxiety and comparable efficacy to alternative forms of phototherapy [35-37]. Notably, integral phototherapy has been observed to utilize less radiant energy from the lamp source, underscoring its enhanced safety and effectiveness [36]. The progressive evolution of phototherapy has yielded a range of secure and efficient treatment modalities for neonatal jaundice, diminishing the necessity for more invasive procedures and enhancing outcomes for affected infants.

Risk-Benefit Analysis

The introduction of phototherapy has yielded significant advancements in the management of neonatal jaundice, particularly in the reduction of the need for exchange transfusions - a procedure historically fraught with risks and challenges, especially in newborns with severe hyperbilirubinemia [36]. This represents a substantial improvement in patient care, offering a safer alternative to a once-dominant intervention. Comparative studies have delved into the effectiveness of intermittent phototherapy (IPT) versus continuous phototherapy (CPT). Notably, findings indicate that IPT is non-inferior to CPT, displaying comparable effectiveness in terms of the rate of fall of bilirubin levels [36]. This suggests that IPT is a viable and equally effective option, providing flexibility in treatment approaches.

One of the notable advantages of phototherapy lies in its ease of administration. This non-invasive treatment can be conveniently delivered at the bedside, streamlining the process for healthcare providers and families [36]. The accessibility and simplicity of administration contribute to the widespread acceptance and adoption of phototherapy as a preferred treatment option. However, as with any medical intervention, careful consideration of potential risks is paramount. Concerns about the potential for oxidative stress in deficient birthweight infants exposed to excessive photons have been raised. This has led to a shift in prescribing phototherapy as a "drug," emphasizing cautious and judicious dosing [36].

Furthermore, the administration of phototherapy is not without side effects, including skin irritation, dehydration, and phototoxicity [36]. These considerations underscore the importance of close monitoring and vigilant management to mitigate potential complications. A nuanced risk-benefit analysis is crucial when employing phototherapy, especially given the potential for overtreatment in cases of mild jaundice. The benefits, including the reduction in exchange transfusion and the non-invasive nature of phototherapy, must be carefully weighed against the risks. This analysis should be conducted on a case-by-case basis, considering the specific clinical needs of the infant and the potential risks and benefits associated with phototherapy. This personalized approach ensures a well-informed and balanced decision-making process in neonatal care.

Innovations and technological advancements

Development of Improved Light Sources

Advancements in phototherapy technology have spurred the development of innovative approaches to treat neonatal hyperbilirubinemia. One notable avenue of exploration is LED phototherapy, which has been subject to studies evaluating its efficacy in addressing unconjugated hyperbilirubinemia. This method utilizes a narrow-band blue LED light, specifically within the 450-470 nm range. The chosen wavelength, notably at 458 nm, aligns with the peak absorption wavelength for bilirubin photoisomerization, demonstrating a targeted and practical approach to treatment [12]. Low-cost HPT systems have been developed to improve accessibility, particularly in developing countries. Examples include the Iranian HPT system and the Brilliance jaundice treatment device, both designed to offer cost-effective solutions for phototherapy in home settings. These systems aim to bridge gaps in healthcare access, particularly in regions where traditional hospital-based phototherapy may be challenging to implement [38].

The integration of LED panels in a phototherapy system represents another noteworthy development. A Vietnamese company has introduced a system that utilizes LED panels, providing a portable solution that can be held on a mother's lap. This unique design promotes breastfeeding and allows the infant to receive continuous phototherapy treatment. Such an approach addresses both the therapeutic needs of the newborn and encourages maternal involvement in the treatment process, fostering a holistic and family-centered approach to care [38]. Researchers have explored wearable phototherapy as a novel approach to advancing innovation. This involves a textile-based wearable organic light-emitting diode (OLED) system, providing a wearable light source for neonatal jaundice treatment. This wearable solution offers a potentially more comfortable and flexible method for administering phototherapy, contributing to developing user-friendly devices that prioritize patient comfort and adherence [39]. Collectively, these advancements in phototherapy technology signify a significant stride toward more efficient, cost-effective, and user-friendly devices for treating neonatal hyperbilirubinemia. By embracing diverse approaches such as LED phototherapy, low-cost home systems, LED panels, and wearable solutions, neonatal care is witnessing transformative changes that aim to enhance treatment accessibility, effectiveness, and patient experience.

Integration of Monitoring and Control Systems

The amalgamation of monitoring and control systems within neonatal jaundice treatment has witnessed noteworthy advancements. Notably, the emergence of HPT devices that can be remotely monitored and wearable devices designed for real-time jaundice detection signifies a pivotal shift in treatment approaches. A pilot study conducted during the COVID-19 lockdown explored HPT for neonatal jaundice and utilized the MIRA-Ginevri phototherapy lamp, showcasing potential efficacy gains. This investigation underscored the critical role of parental compliance and confidence in independently operating the equipment without constant supervision. Moreover, it emphasized the imperative for bilirubin monitoring, accentuating the potential viability of HPT as an effective treatment option [40].

A distinct innovation in this realm is introducing a neonatal wearable device for colorimetry-based real-time jaundice detection, concurrently enabling vital sign monitoring. This technological marvel facilitates continuous bilirubin level monitoring during phototherapy, effectively addressing the challenge of precision in bilirubin monitoring throughout treatment. The study associated with this device demonstrated the feasibility of a synergistic treatment approach incorporating an integrated monitoring system [41].

In addition, a groundbreaking proposal involves a novel light source-based phototherapy for neonatal jaundice utilizing a textile-based wearable OLED system. This wearable phototherapy approach signifies a substantial leap forward in seamlessly integrating monitoring and treatment for neonatal jaundice [39]. These developments, encompassing HPT and wearable monitoring devices, signify notable strides in the integration of monitoring and control systems for neonatal jaundice treatment. They promise enhanced efficacy, increased parental involvement, and real-time monitoring capabilities.

Advances in Home-Based Phototherapy

The adoption of home-based phototherapy has ushered in significant advantages, spanning cost-effectiveness, improved maternal-infant bonding, reduced parent-infant separation, the advent of wearable phototherapy, and fruitful stakeholder alliances. Firstly, in terms of cost-effectiveness, a randomized controlled trial has validated home-based phototherapy as a financially prudent alternative to hospital-based phototherapy. The trial revealed a substantial cost reduction of €819 (71%) per patient, primarily attributable to diminished inpatient care costs [42]. This underscores the potential economic benefits of home-based phototherapy, presenting a viable and cost-efficient treatment option.

Beyond financial considerations, home-based phototherapy has demonstrated multifaceted benefits. Numerous studies have corroborated that it enhances access to care, fosters maternal-infant bonding, and augments patient satisfaction [43]. The significance of these outcomes extends beyond clinical efficacy, acknowledging the broader impact on the well-being of both the infant and the caregiver. A pivotal aspect of home-based phototherapy is its role in reducing parent-infant separation, a factor that holds considerable weight in mitigating maternal postpartum psychoemotional distress [40]. The ability to provide treatment within the familial context not only contributes to medical care but also acknowledges the psychosocial dynamics of the family, thereby promoting a more holistic approach to neonatal care.

In tandem with these advancements, researchers have proposed a groundbreaking wearable phototherapy solution using a textile-based OLED system. This innovative approach holds promise in providing a comfortable and portable means of administering phototherapy, potentially enhancing patient compliance and convenience [39]. Furthermore, the success of HPT programs has been closely tied to collaborative efforts involving insurance carriers, device manufacturers, and hospitals. These stakeholder alliances have played a pivotal role in establishing and sustaining effective HPT initiatives, showcasing a model that can be replicated by other medical institutions [44].

Challenges and limitations

Compliance and Duration of Treatment

The duration of phototherapy for neonatal hyperbilirubinemia is contingent upon factors such as the infant's age, bilirubin levels, and response to treatment. In accordance with guidelines from the American Academy of Pediatrics (AAP), discontinuation of phototherapy is recommended when an infant readmitted for hyperbilirubinemia reaches a level of 13-14 mg/dL, having initially presented with a level of 18 mg/dL or more [22]. While there are no specific directives regarding when to cease phototherapy, the presence of hemolysis and the infant's age play crucial roles in determining the duration. The necessity for phototherapy can vary widely, ranging from as brief as 24 hours to an extended period of 5 to 7 days, contingent upon individual cases [22]. The efficacy of phototherapy hinges on factors such as the distance between the lamps and the infant, the intensity of the light source, and the total light dose received. Maximizing the skin surface area exposed to phototherapy is also recognized as a strategy to enhance treatment outcomes [22].

The decision to commence or conclude phototherapy is grounded in the newborn's age and total serum bilirubin level. Generally, highly effective for newborn jaundice and associated with minimal side effects [45], phototherapy is administered to expose the baby's skin to maximal light. Typically, treatment cessation occurs when bilirubin levels reach a safe threshold, which commonly takes one to two days [45]. If jaundice persists, intensified phototherapy may involve an augmentation in light intensity or the simultaneous use of an alternative light source, such as a light blanket [45]. The duration of phototherapy for neonatal hyperbilirubinemia is determined on a case-by-case basis, considering the infant's age, bilirubin levels, and treatment response. The AAP guidelines provide specific benchmarks for discontinuation, underscoring the generally practical nature of the treatment with minimal side effects.

Resource Limitations in Low-Income Settings

The challenges surrounding the effective implementation of phototherapy in neonatal care are particularly pronounced in low-resource settings, where limited accessibility poses a significant barrier. These regions often lack the specialized equipment and trained personnel necessary for properly administering phototherapy, rendering it largely inaccessible [46]. The consequent impact is a compromised ability to provide timely and effective treatment for neonatal hyperbilirubinemia. Furthermore, issues related to the improper installation and maintenance of phototherapy devices exacerbate the challenges faced in low-resource settings. Without adequate installation and regular maintenance, these devices may not function optimally, leading to suboptimal treatment outcomes. It underscores the importance of simple adjustments and regular upkeep in ensuring the effectiveness of phototherapy interventions [46].

Cost and resource constraints further compound the challenges in low-income settings, particularly concerning the affordability of phototherapy equipment, especially those utilizing LED lights. Limited financial resources hinder the acquisition and maintenance of these devices, posing a substantial impediment to implementing effective neonatal jaundice management [46]. The inadequacy of screening and monitoring tools in these settings also contributes to the challenge. The absence of non-invasive screening tools and essential therapeutic equipment impedes accurate screening, treatment, and monitoring of neonatal hyperbilirubinemia. This deficiency compromises the ability to identify and address the condition in a timely and effective manner [46].

Education and training gaps among healthcare providers in low-income settings present another significant hurdle. To ensure the proper utilization of phototherapy and accurate assessment of neonatal jaundice, there is a crucial need for comprehensive education and training programs for healthcare personnel in these regions [47]. Addressing these multifaceted challenges requires a comprehensive approach, encompassing the development of cost-effective phototherapy solutions, training initiatives for healthcare professionals, and efforts to improve access to essential medical equipment in low-income settings. This holistic strategy is essential to bridge existing gaps and enhance neonatal care in resource-constrained environments.

Potential Long-Term Effects

Phototherapy stands as a recognized and effective treatment for neonatal hyperbilirubinemia, yet it is imperative to acknowledge the potential short-term and long-term adverse effects linked with its application. In the short term, adverse effects encompass skin rashes, dehydration, and disruptions in electrolyte balance. The spectrum of long-term adverse effects extends to concerns such as DNA damage, elevated cancer risk, and potential neurodevelopmental delays [13]. Notably, a study has posited an association between phototherapy and the occurrence of late-onset solid tumors, including those affecting the brain and central nervous system [48]. Nevertheless, the precise mechanism underpinning these adverse effects remains elusive, emphasizing the necessity for more comprehensive studies aimed at unraveling these intricacies and optimizing innovative therapeutic approaches in the future [13].

## Conclusions

In conclusion, this comprehensive review of the evolution of phototherapy for neonatal hyperbilirubinemia has illuminated vital aspects crucial for advancing neonatal care. The historical progression from early recognition of neonatal jaundice to the development of diverse phototherapy modalities underscores the dynamic nature of treatment strategies over time. The physiological intricacies of bilirubin metabolism in neonates, with a focus on delayed hepatic maturation, emphasize the vulnerability of newborns to hyperbilirubinemia. Phototherapy, through its diverse modalities, consistently demonstrates efficacy in reducing unconjugated bilirubin levels, thus mitigating the risk of bilirubin-induced neurotoxicity. The implications for clinical practice are profound, offering healthcare professionals valuable insights into tailoring treatment strategies based on the severity of jaundice and individual neonatal characteristics. Furthermore, integrating advanced monitoring and control systems enhances precision and safety in delivering phototherapy. Recommendations for future research highlight the need for in-depth investigations into long-term outcomes and potential neurodevelopmental effects, exploration of adjunctive therapies, and addressing resource limitations for global access to effective neonatal care. In essence, this review contributes to the ongoing refinement of neonatal care practices, fostering a deeper understanding of neonatal hyperbilirubinemia and its treatment landscape.
